# Recurrent, Severe Aphthous Stomatitis and Mucosal Ulcers as Primary Manifestations of a Novel *STAT1* Gain-of-Function Mutation

**DOI:** 10.3389/fimmu.2020.00967

**Published:** 2020-05-28

**Authors:** Melinda Erdős, Eszter Jakobicz, Beáta Soltész, Beáta Tóth, Zsuzsanna Bata-Csörgő, László Maródi

**Affiliations:** ^1^Department of Infectious and Pediatric Immunology, Faculty of Medicine, University of Debrecen, Debrecen, Hungary; ^2^PID Clinical Unit and Laboratory, Department of Dermatology, Venereology and Dermatooncology, Semmelweis University, Budapest, Hungary; ^3^St. Giles Laboratory of Human Genetics of Infectious Diseases, Rockefeller University, New York, NY, United States; ^4^Insitute of Laboratory Medicine, University of Szeged, Szeged, Hungary; ^5^Department of Dermatology and Allergology, Albert Szent-Györgyi Medical Center, University of Szeged, Szeged, Hungary

**Keywords:** mucosal ulcers, STAT1, gain-of-function mutation, IL-17-mediated immunity, mucocutaneous candidiasis

## Abstract

Chronic mucocutaneous candidiasis (CMC) characterized by persistent and recurrent Candida infection of the skin, nails, and the mucosa membranes has been proposed as the major infectious phenotype in patients with gain-of-function mutation of signal transducer and activator of transcription 1 (STAT1) 1. However, viral infections caused mostly by herpesviruses, and a broad range of autoimmune disorders may also be part of the clinical phenotype. We report here on a 31 years old female patient suffering from severe mucosal aphthous mucositis and ulcers and recurrent herpes simplex for decades. We found a previously unknown heterozygous sequence variant in *STAT1* (c.1219C>G; L407V) affecting the DNA-binding domain of the protein in the patient and her 4 years old daughter. We found this mutation gain-of-function (GOF) by using immunoblot and luciferase assays. We detected low proportion of IL-17A-producing CD4+ T cell lymphocytes by using intracellular staining and flow cytometry. Candida-induced secretion of IL-17A and IL-22 by mononuclear cells from the patient was markedly decreased compared to controls. These data suggest that the novel mutant allele may result in impaired differentiation of CD4+ T cells to CD4+/IL-17+ cells. The clinical phenotype of the disease in this patient was unique as it was dominated primarily by severe aphthous stomatitis and ulcerative esophagitis and only partly by typical CMC resulting in diagnostic delay. We suggest that patients with severe recurrent aphthous stomatitis and esophagitis should be evaluated for *STAT1* GOF mutation. Based on the broad clinical spectrum of the disease, we also suggest that CMC and CMC disease may not be an appropriate term to define clinically *STAT1* GOF mutation.

## Introduction

Impaired interleukin (IL)-17 mediated T cell immunity has been described to associate with chronic mucocutaneous candidiasis (CMC) (1–3). The most common genetic cause of CMC is thought to be gain-of-function (GOF) mutation in signal transducer and activator of transcription 1 (*STAT1*) affecting predominantly the coiled-coil domain (CCD) and less frequently the DNA-binding domain (DBD) (4–6). Importantly, bacterial and viral disease are also common in patients with *STAT1*-GOF occurring in 74 and 38%, respectively, and viral infections, herpesviruses in particular, preferably cause diseases on the surface barrier (6, 7).

Candida species, especially *C. albicans* reside on body surfaces of healthy individuals as innocent commensals. This symptomless commensalism, also referred to as colonization, may proceed to symptomatic candidiasis especially in patients with HIV infection and AIDS, and in those who have genetically impaired CD4+CD17+ T cell immunity (7). Mucosal candidiasis is commonly seen in patients with aphthous stomatitis and mucosal ulcer, but it is challenging to define if epithelial damage is caused primarily by Candida or, alternatively, fungal superinfection is a consequence of impaired barrier structure and function (8, 9).

We report here on a female patient who has been suffering primarily from severe, recurrent and persistent aphthous stomatitis since infancy. It was only at age 25 when genetic analysis was performed and revealed a novel *STAT1* GOF mutation (c.1219C>G; L407V). An impaired CD4+IL17+ T cell differentiation and function was also found. The patient was diagnosed with autoimmune mucositis and CMC was not considered as a primary disease-causing entity leading to remarkable delay in molecular diagnosis. We propose that patients with unexplained chronic aphthous stomatitis and esophagitis may have *STAT1* GOF mutation. We also suggest that *STAT1* GOF mutation is a more accurate disease term than CMC because of the broad and heterogeneous clinical manifestations including non-fungal infections, autoimmune disease, endocrinopathies, and rarely, cerebral aneurisms (6).

## Case Reports

### Patient 1

This 31-year-old Hungarian female patient was born at term with 2,380 g birth weight and 47 cm length. During the time Hungary was polluted from air by the radiation originated from Chernobyl in May 1986 her mother spent a full day outdoor and developed an undiagnosed disease with fatigue, dizziness and diarrhea lasting for a week. Otherwise the pregnancy was uneventful, and the patient was born with no complication. The patient's father developed bladder cancer at age 41 which was treated successfully by surgery and chemotherapy. The umbilical cord of the patient detached 13 days after birth and local infection of the stub by Candida was diagnosed and treated successfully with local agents. She was immunized with Bacille-Calmette-Guérin vaccine at 3 days after birth and had seropurulent discharge from the site of injection at 6 and 9 months of age, respectively, for a few days. She received other childhood immunizations including diphtheria, pertussis, tetanus, poliomyelitis (Sabin vaccine), and one shot of measles-mumps-rubella vaccine without complication. Remarkably, she developed fever of 39–40 °C after each shot. At 7 months of age she developed aphthous stomatitis which recurred monthly at the beginning and more frequently later on but oral candidiasis was not visible. The second episode of aphthous stomatitis at age 3 required hospitalization and this time oral candidiasis was also diagnosed and she was treated with local nystatin and metronidazole. Pharyngeal and stool cultures yielded *C. albicans*. Sedimentation rate was 9 mm/h, platelet number, 122.5 Giga/L, serum iron and zinc levels were 5.5 and 7.43 μM, respectively. Tuberculin skin test evoked an 8 × 8 mm erythematous nodule, HIV serology and direct and indirect Coombs tests were negative. T cell number evaluated with rosetta test was 55% of lymphocytes (normal, 75–80%), T cell proliferation assay showed 35% blasts (normal, 40–45%), serum IgM, 1,15 g/L, IgA, 1,12 g/L, IgG, 12,3 g/L), complement hemolytic activity and granulocyte respiratory burst activation (nitroblue tetrazolium reduction and superoxide anion generation) were normal. She was discharged with the clinical diagnosis of undefined “Immunodeficiency.” At age 5 she developed chickenpox, received hyper immune globulin and oral acyclovir and developed only about 20 vesicles and a mild course of the disease. Later on, however, she developed herpes simplex each year for 4–5 years, usually in August, and from age 24 she had recurrent herpes simplex every other year for 3 times mostly in the back and once in the lower abdomen with lesions of 8–10 cm size. At age 7 she was admitted to hospital with high fever and cough. WBC was 19,3 Giga/L, sedimentation rate, 98 mm/h, platelet number, 224 Giga/L, hemoglobin, 122 g/L, serum IgM, A, and G were 0.87, 1.14, and 20.8 g/L, respectively. Chest X-ray showed signs of bacterial pneumonia and did not suggest fungal etiology. Culture of urine, stool, throat scrub, nasal scrub, sputum and stool did not yield bacterial or candida pathogen. Serology and sputum culture for Mycoplasma, Chlamydia, Legionella, and *P. jiroveci* gave negative results. Treatment with various combinations of vancomycin, ceftazidime, brulamycin, clindamycin, and monobactam antibiotics and diflucan, the latter given because of the prolonged antimicrobial treatment, resulted in recovery from the severe, lower respiratory tract infection. At 13 years of age she met a bicycle accident and developed multiple fractures in her left tibia and fibula. Even after normal bone healing her left lower extremity remained 2 cm shorter than the right one. Since the first hospital admission she was scheduled for yearly immunology checkup and a decrease in the ratio of CD3+ and CD4+ T lymphocytes and occasionally an increase in the B lymphocytes was detected. Autoimmune and allergy serology tests gave negative results, only ASCA IgA and IgG was somewhat elevated (26–67 U/l) over the years. During her school age she was no more susceptible to viral respiratory or gastrointestinal infections than her schoolmates. She was somewhat even more resistant to community infections including flu compared to her schoolmates. Remarkably, however, when she had infection occasionally, she always had high fever and when she received antibiotic treatment, she always developed oral stomatitis. Despite the lack or recurrent infections her weight percentile remained below 3 and she had microcytic anemia all over the time. Since her school age she has had persistent and recurrent eczema in the crook of the arm, the ham, the neck and the eyelids. At 17 years of age she was hospitalized for high fever, bilateral cervical lymphadenopathy, severe stomatitis with deep ulcers and difficulty eating and swallowing. Epstein-Barr virus serology revealed past infection with negative serum anti-VCA IgM and positive anti-EBV nuclear antigen. Adenovirus and cytomegalovirus serology gave negative results. Elevated liver enzymes were first detected at age 18 and remained slightly elevated afterwards. At age 18 she developed gastroenteritis and high fever which was attributed to Campylobacter species infection. At the age 21 chest X-ray was performed because of cough and rales on both sides and revealed patchy alveolar infiltrations around both hilus and fine interstitial infiltrations in both lower lobes. Inactive, 5–6 mm-sized, homogenic infiltrations were found in the upper lobes. Amoxicillin-clavulanic acid, clarithromycin and methylprednisolone treatment resulted in partial relief of cough, and the pneumonia finally resolved for levofloxacin and amoxiclav. Bronchoscopy and mycobacterial culture gave negative results. Calcification of the previously found upper lobar lesions was detected by X-ray. A host of autoimmune and allergy serology tests gave negative results and the etiology of the lung disease remained undefined. *Campylobacter coli* infection was diagnosed again at age 22. Between 19 and 25 years of age when she was a medical student, oropharyngeal and esophageal ulcers developed more and more frequently and at age 25 she did not become symptomless and had daily fever without defined infectious disease. During this period, she underwent esophago-gastro-duodenoscopy 9 times and colono-ileoscopy twice. These examinations revealed severe ulcerous lesions all over the length of the esophagus once with Candida patches, scars and narrowing at several locations. In the stomach and duodenum lymphatic stasis was detected. Colono-ileoscopy ruled out inflammatory bowel disease; however, histology of the esophagus at the peak of the symptoms complied with the manifestation of the Crohn's disease in the upper gastrointestinal tract, and no histology could detect any sign of fungal infection. At age 25 she underwent surgery for gluteal abscess and treated with amoxiclav and ciprofloxacin empirically.

**Figure 1:**
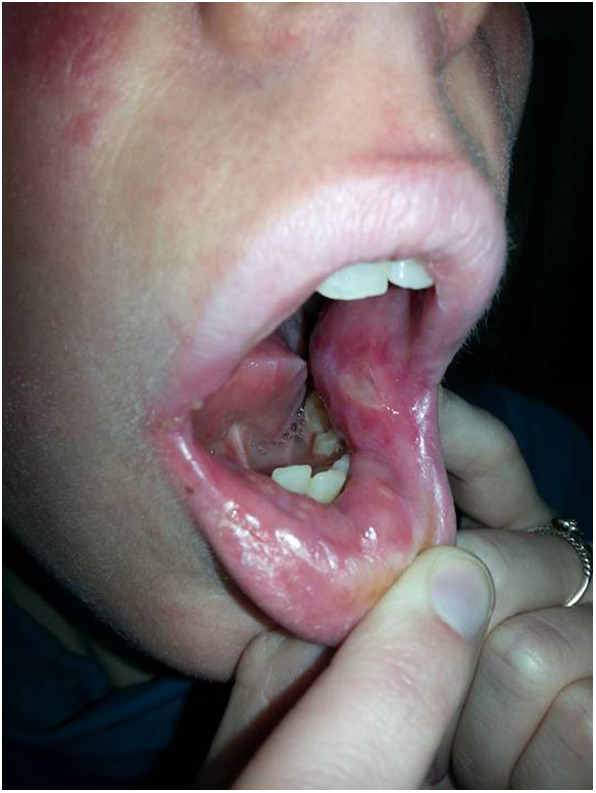
Painful, deep ulcer in the left buccal mucosa at age 26.

We first saw the patient at age 25 during the episode what the patient defined as the most severe attack of oropharyngeal ulceration (Figure 1). Her weight was 37 kg and her height was 167 cm. Several deep ulcers of 5–14 mm in the buccal mucosa were seen and the patient could only swallow liquid. The facial skin was covered with papulopustulosus lesions of rosacea mostly in the nasolabial and maxillary areas. Chest CT scan revealed miliary lesions in the left apical and posterior segments. Blood culture yielded alfa-hemolytic Streptococci and buccal swab culture was positive for *S. aureus*. Bronchofiberoscopy showed atrophic, fragile bronchial mucosa but mucosal swab culture gave negative result. Coccidioides serology on admission and 8 months later revealed “TP” antibodies in the serum. CMC was thought and targeted *STAT1* sequencing was performed which revealed a novel sequence variant affecting the DBD. Functional analysis suggested GOF mutation (see Results). Parenteral acyclovir, clindamycin and fluconazole treatment was started, but her condition did not improve. Her treatment was switched to a combination of Caspofungin and low dose (30 mg/day) prednisone with local agents including nystatin, amphotericin B, xanthoflavin, lidocaine, and ascorbic acid was applied. Her condition improved in a week and full oral food intake and calorization could be started. The patient's general condition and appetite improved, and she started to gain weight. After she became free of ulcer, fluconazole maintenance therapy at a dose of 150 mg/week and prednisone at a 5 mg maintenance dose were continued for a few weeks. She was without therapy and remained stable for 1 year. At 27 year of age she became pregnant and in the second semester she developed upper respiratory tract disease and took amoxiclav for 7 days which precipitated stomatitis and ulcers. Prednisone at 10 mg dose was effective for the ulcers but in the third semester soor appeared on the oral mucosa and *C. glabrata* was detected from the vaginal scrub. At this age she experienced onychomycosis in her right thumb which proved to be recalcitrant to local therapy. She started again fluconazole at a dose of 2 × 150 mg/week Her mucositis was well controlled by treatment with local antifungals and 10 mg, and later on 5 mg per day prednisolone. Otherwise the pregnancy was uneventful, and in January 2016, she gave birth to a girl of 2,740 g and 47 cm (Patient 2, see below). She went off medications and for more than a year was well. At 29 years of age she again developed oropharyngeal and esophageal ulcers that was treated with steroids and fluconazole which resulted in rapid improvement. Over the past years she has had transient and mild oral aphtha and esophagitis responsive to short courses of prednisolone (maximal 30 mg per day) and fluconazole (maximal 2 × 150 mg weekly). She has been on 5 mg per day prednisolone and 150 mg per week fluconazole maintenance therapy.

### Patient 2

**Figure 2:**
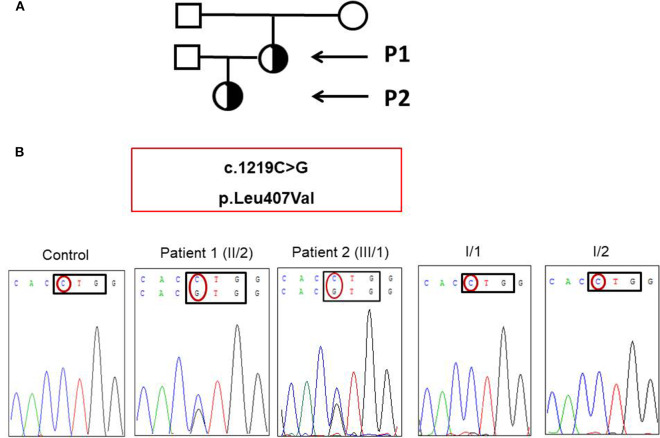
**(A)** Pedigree of patient 1 and patient 2 (black arrows). **(B)** Novel heterozygous mutation affecting the DNA binding domain of *STAT1* was detected in the patient and her daughter.

The baby of Patient 1 was breastfed for 6 months and she has had only mild gluteal and oral candidiasis after birth (Figure 2A). She received routine immunization, and in addition, varicella, meningococcus C and tick encephalitis vaccines without complication. She had fluconazole responsive oral candidiasis treated for 2 months at age 1 year. She started kindergarten at 112 year of age, and she had not developed Candida infection again but had 16 consecutive episodes of purulent otitis media and treated with several antibiotics over the past year. She has also had recurrent upper respiratory tract viral infections and underwent adenectomy at 212 years of age. Despite the recurrent infections her growth and development has been appropriate for age. Genetic analysis revealed the same *STAT1* GOF mutation as found in her mother (See Results).

## Methods

### Genetic Analyses

Genomic DNA from the patient and her relatives were isolated with the Gen Elute Blood Genomic DNA kit (Sigma-Aldrich, St Louis, Missouri, USA). Mutations were analyzed by amplifying exons and flanking intronic regions of *STAT1* by PCR. The PCR primers and sequencing primers are available on request. Amplicons were sequenced with the Big Dye Terminator cycle sequencing kit (Applied Biosystems, Foster City, California, USA). And targeted regions were analyzed by an ABI 3,130 capillary sequencer (Applied Biosystems). Sequence variants were determined compare to reference sequence, GenBank accession no. ENST00000361099 of the STAT1 cDNA to identify the position of mutations. Mutations are nominated according to Dunnen and Antonarakis (9). The pathogenicity of missense variant identified was investigated by *in silico* analysis using SIFT and PolyPhen2 software. According to the PolyPhen2 score the amino acid substitution is predicted to be “probably damaging” if the score is >0.908. According to the SIFT score the amino acid substitution is predicted to be “deleterious” if the score is <0.05.

### Cell Isolation

Peripheral blood mononuclear cells (PBMCs) were isolated by gradient centrifugation (Ficoll-Paque PLUS, GE Healthcare Bio-Science AB, Uppsala, Sweden) and resuspended in DMEM (Sigma-Aldrich Inc., St Louis, Missouri, USA) supplemented with 10% heat-inactivated FBS (Gibco) and 1% Pen/Strep (Sigma- Aldrich). Adherent monocytes were removed by incubation for 3–4 h at 37°C.

### Western Blot

Generation of EBV-transformed lymphoblasts was carried out as previously described (10). EBV-B cells were stimulated by incubation with IFN-γ (PeproTech, London, UK, Eu). We assessed dephosphorylation by stimulation with IFN-γ and then incubation with staurosporine (Sigma-Aldrich) and nuclear proteins were extracted with Protein Fractionation Kit (Thermo Scientific, Rockford, IL, USA) and subjected to immunoblot analysis. We used antibodies against phosphorylated STAT1 (pY701, BD, San Jose, CA, USA), STAT1 (C-24, Santa Cruz, Dallas, Texas, USA), and lamin B1 (Santa Cruz, Dallas, Texas, USA).

### Generation of IL-17-Producing T Cells

Non-adherent blood cells were cultured in anti-CD3 antibody-coated plates (Miltenyi Biotec, Bergisch Gladbach, Germany, Eu) in the presence of interleukin (IL)-23, IL-6, IL-1β, and transforming growth factor (TGF)-β1 (Pepro Tech). After 2 days, cells were restimulated with IL-2 (PeproTech), together with IL-1β, IL-23, IL-6, and TGF-β1. After 5 days, the cells were stimulated with phorbol 12-myristate 13-acetate (PMA; Sigma- Aldrich) and ionomycin (IMC; Sigma-Aldrich) in the presence of GolgiPlug (Sigma-Aldrich) for flow cytometry analysis and for ELISA without GolgiPlug. The detailed protocol was described earlier (10).

### Flow Cytometry

For flow cytometry analysis, the cells were incubated for the surface labeling with allophycocianin (APC)-conjugated anti-hCD4 IgG_1_ monoclonal antibody (mAb) (BD, San Jose, CA, USA) and Peridinin chlorophyll (PerCP)-conjugated anti-hCD3 IgG_1_ monoclonal antibody (BD). After fixation the cells were stained with phycoerythrin (PE)-conjugated mouse anti-human IL-17A IgG_1_mAb (R&D Systems, Minneapolis, MN, USA) and fluorescein isothiocyanate (FITC)-conjugated anti-human IL-22 IgG_1_mAb (R&D Systems) antibodies. Cells were analyzed with an Accuri C6 flow cytometer (BD).

### Elisa

*C. albicans* (ATCC 10231) was maintained and heat-inactivated as described earlier (10). Candida-induced secretion of IL-17A and IL-22 was determined by ELISA. The supernatants were harvested after stimulation of cells with PMA and ionomycin and after *in vitro C. albicans* stimulation. Human IL-17 Quantikine ELISA Kit and Human IL-22 Quantikine ELISA Kit (R&D Systems) were used according to the manufacturer's instructions.

### Luciferase Reporter Assay

STAT1 mutations were generated by site-directed mutagenesis (QuikChange Site-Directed Mutagenesis kit, Stratagene, La Jolla, CA, USA). We transfected the STAT1-deficient U3C fibrosarcoma cells with 100 ng/well reporter plasmids (Cignal GAS Reporter Assay, SA Biosciencies) and plasmids carrying the c.1219C>G (L407V), c.821G>A (R274Q), c.2117T>C (L706S), and wild type allele of *STAT1* or a mock vector in the presence of Lipofectamine LTX (Invitrogen). The transfected cells were then stimulated with IFN-γ (10 and 1,000 U/ml) and analyzed by Dual Luciferase assay system (Promega).

## Results

### Mutational Analysis of *STAT1*

We found a novel heterozygous c.1219C>G mutation of *STAT1* located in exon 14 of the patient and her daughter (Figure 2B). This mutation was not found in the samples of the mother and father. The parents have had no recurrent and persistent mucosal disease. *In silico* predictions (SIFT and PolyPhen2) of the novel *STAT1* variant showed that this mutation was predicted to be “deleterious” and “probably damaging” with a score of 0.00 (SIFT), score of 0.999 (HumDiv, PolyPhen2; sensitivity: 0.14; specificity: 0.99), and score of 0.980 (HumVar, PolyPhen2; sensitivity: 0.57; specificity: 0.94), respectively. This mutation was not found in 50 healthy controls by using bidirectional DNA sequencing.

### Gain-of-Phosphorylation of Mutant STAT1

**Figure 3:**
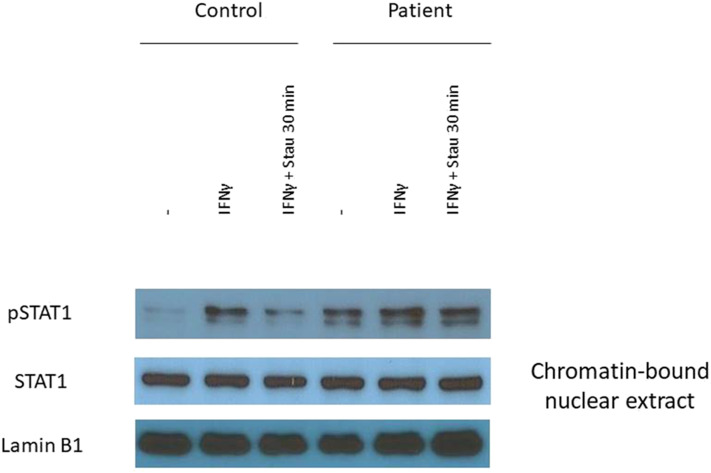
The L407V amino acid change in STAT1 results in gain-of-phosphorylation. The expression of pSTAT1 and STAT1 were assessed by immunoblot. EBV-B lymphocytes were stimulated with IFN-γ for 30 min. To determine the inhibition of dephosphorylation we added tyrosine inhibitor staurosporine into the cell suspension for 30 min.

We prepared EBV-B lymphoblasts from the patient and a healthy control. After incubation with IFN-γ we detected increased STAT1 phosphorylation in nuclear extracts of patient's EBV-B cells compared to that of control cells (Figure 3). We determined the dephosphorylation of STAT1 after stimulation with tyrosine kinase inhibitor staurosporine, and we found that dephosphorylation was impaired in the patient carrying the mutant allele (Figure 3). These data suggested GOF and loss-of-dephosphorylation of STAT1 due to the c.1219C>G mutation.

### The New c.1219C>G Mutation of *STAT1* Results in GOF for γ-Activated Factor (GAF)-Dependent Cellular Responses

**Figure 4:**
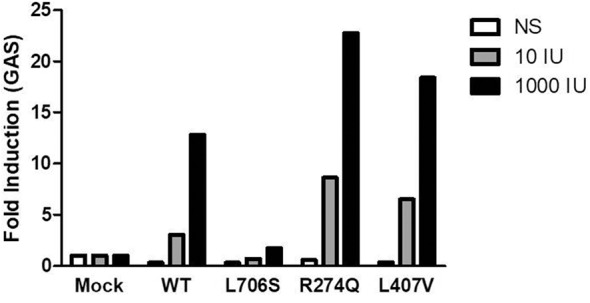
Fold induction of γ-activated sequence (GAS)-dependent reporter gene transcription activity was assayed after stimulation with 10 IU/ml or 1,000 IU/ml IFN-γ. The R274Q allele used as positive control, the L706S allele as negative control, and the L407V as a new mutant allele were transfected to U3C cells. The data represent 2 independent experiments performed in triplicate.

Plasmids with the new mutant alleles were created by site-directed mutagenesis and the position of the mutation was confirmed by Sanger sequencing. U3C cells, that lack of endogenous STAT1 were transfected with the new c.1219C>G (L407V) allele, a CMC-causing c.821G>A (R274Q) allele serving as positive control, an MSMD-causing c.2117T>C (L706S) allele serving as negative control, and the wild type allele of *STAT1*. The luciferase activity of the reporter gene was measured under the control of the γ-activated sequence (GAS) promoter. After 1,000 IU/ml IFN-γ stimulation two times stronger luciferase activity was measured in U3C cells transfected with the novel L407V allele and GOF R274Q allele than in cells with the wild type allele. These data suggested that the new allele is GOF for GAF-dependent cellular responses to IFN-γ (Figure 4).

### Impaired Differentiation of CD4+ T Cells Into IL-17+ Cells in the Patient

We determined the percentage of CD3+/IL-17+ and CD3+/IL-22+ and CD4+/IL-17+ and CD3+/IL-22+ T cells after incubation with PMA and IMC as described. Cells from the patient with heterozygous L407V allele showed markedly decreased proportion of IL-17+ and IL-22+ T cells among CD3+ and CD4+ cells compared to healthy controls (Figure S1).

### Secretion of IL-17A and IL-22 Cytokines

Cytokine concentrations were detected in the supernatant of PBMCs after stimulation with heat-killed Candida and from the supernatant of IL-17+ T cells after stimulation with PMA and IMC. PBMCs and T cells from the patient secreted decreased amounts of IL-17A and negligible amount of IL-22 compared to controls (Figure S2).

## Discussion

The unique feature of patient 1 was the chronic damage of mucous membranes of the oropharynx and esophagus without visible and detectable candidiasis most of the time. This report highlights the remarkable lack of awareness on PID in the medical community and emphasizes the ongoing importance of physician education campaigns like the J Project (11, 12). Clinicians should be more aware of the heterogenous phenotype of *STAT1* GOF mutation. In patient 1 the “misleading” manifestation of the disease was the massive involvement of oropharyngeal mucosa and recurrent and persistent aphthous stomatitis and esophageal ulcers for decades. Aphthous stomatitis could be a complication of CMC. It is possible that once Candida causes damage to the mucous membranes additional host defenses like evasion of phagocytes and serum factors may control the overgrowth of fungi. In patient 1 several mucosal biopsies were performed, and the samples from the esophagus were negative for fungal infection, but positive for EBV genomic markers by PCR suggesting further the critical role of herpes viruses in the pathology of mucositis (7). *STAT1* GOF mutation may result in massive inflammation at mucosal surfaces resulting in aphthous stomatitis and ulcers which may be independent of candida mucositis; moreover, they might be manifestations of Crohn's disease in the upper gastrointestinal tract, as an autoimmune complication of STAT1 GOF mutation. The fact that the mucosal ulcers are responsive to prednisone suggest a role of immune activation in the pathogenesis. From the clinical point of view, we believe that mutational analysis of *STAT1* and enumeration and characterization of Th17 cells is warranted in patients with recurrent and progressive oral and esophageal mucositis without solid clinical and immunology diagnosis.

Meyer et al. (13) proposed that the single L407V amino acid change, did not affect the subcellular resting distribution of STAT1 or nuclear clearance after interferon-γ stimulation. It was suggested that changing from leucine to isoleucine or valine may not affect the hydrophobic nature of the side chains (13). In contrast, we found impaired development of IL-17+ and IL-22+ T cells associated with the higher susceptibility to Candida infections in patient 1. These findings were in concert with the low amount of IL-17+ and IL-22+ T cells and small concentrations of IL-17A and IL-22 cytokines released by Candida-exposed mononuclear blood cells. Our data suggest that this novel mutation which may not interfere with transport mechanisms such as import-export of STAT1 is still pathogenic. Higher activity of the luciferase reporter gene under the control of the GAS promoter analyzed in U3C cells transfected with L407V allele after IFN-γ stimulation compared to those transfected with the wild type or MSMD-causing alleles confirmed this hypothesis.

In conclusion, the new mutant allele is GOF and adds to the list of *STAT1* GOF mutations (14). The unique, but not exceptional clinical phenotype in this patient remains to be elusive. Heterozygous *STAT1* GOF patients show an unexpectedly broad clinical phenotype (6, 14). Environmental factors and effects of modifying genes involved in phenotypic manifestation of *STAT1* L407V mutation may have a role.

## Data Availability Statement

The raw data supporting the conclusions of this article will be made available by the authors, without undue reservation, to any qualified researcher.

## Ethics Statement

The studies involving human participants were reviewed and approved by Ethics Committee of the University of Debrecen. Written informed consent to participate in this study was provided by the participants' legal guardian/next of kin.

## Author Contributions

All authors listed have made a substantial, direct and intellectual contribution to the work, and approved it for publication. ME: diagnostic workup, evaluation of data, and writing the paper. EJ: design of diagnostic setup and follow the patient. BS and BT: performing laboratory experiments. ZB-C: designed the experiments, follow up and treatment the patient, and writing the paper. LM: study design, treatment coordination, differential diagnostic workup, treatment the patient, writing the paper, and coordinating research.

## Conflict of Interest

The authors declare that the research was conducted in the absence of any commercial or financial relationships that could be construed as a potential conflict of interest.
