# Supplementary material for: Recurrent, Severe Aphthous Stomatitis and Mucosal Ulcers as Primary Manifestations of a Novel STAT1 Gain-of-Function Mutation
Source: Front Immunol. 2020 May 28;11:967. doi: 10.3389/fimmu.2020.00967 (PMC7270203; doi:10.3389/fimmu.2020.00967)
Supplement: Supplementary file 1 [file Table_1.DOCX]

**SUPPLEMENTARY** **FIGURE LEGENDS**

**SFigure 1** Ex vivo analysis of development of IL-17+ and IL-22+ T cells from the patient and control. Percentages of CD3+/IL-17+, CD3+/IL-22+, CD4+/IL-17+ and CD4+/IL-22+ cells were determined by flow cytometry after 6 h incubation with phorbol 12-myristate 13-acetate and ionomycin.

**SFigure 2** Concentrations of IL-17A and IL-22 cytokines by blood mononuclear cells and IL-17+ T cells from patient and healthy control were measured by enzyme-linked immunosorbent assays (ELISA). Candida stimulation with heat-killed *C. albicans* (10^5^). PMA+IMC: treatment with phorbol 12-myristate 13-acetate (50 ng/ml) and ionomycin (10^-5^ M) for 48h. C: control; P: patient.
