# Supplementary figures and images for: Recurrent, Severe Aphthous Stomatitis and Mucosal Ulcers as Primary Manifestations of a Novel STAT1 Gain-of-Function Mutation
Source: Front Immunol. 2020 May 28;11:967. doi: 10.3389/fimmu.2020.00967 (PMC7270203; doi:10.3389/fimmu.2020.00967)

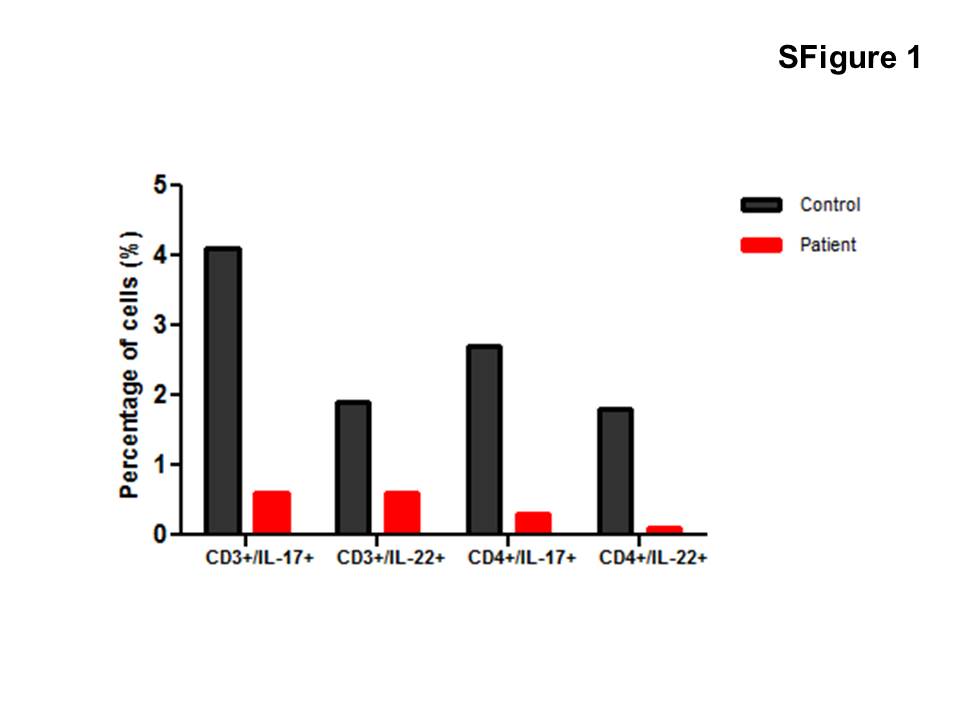

Supplement: Supplementary file 2 [file Image_1.JPEG]

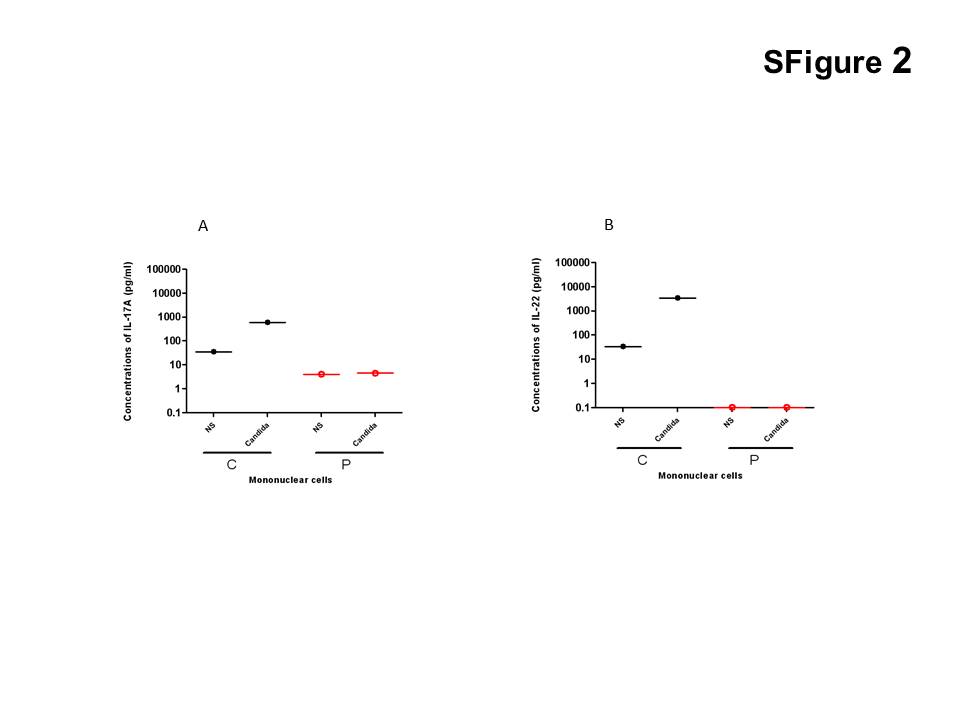

Supplement: Supplementary file 3 [file Image_2.JPEG]
